# Degradation of the Escherichia coli Essential Proteins DapB and Dxr Results in Oxidative Stress, which Contributes to Lethality through Incomplete Base Excision Repair

**DOI:** 10.1128/mbio.03756-21

**Published:** 2022-02-08

**Authors:** Charley C. Gruber, Vignesh M. P. Babu, Kamren Livingston, Heer Joisher, Graham C. Walker

**Affiliations:** a Department of Biology, Massachusetts Institute of Technology, Cambridge, Massachusetts, USA; Massachusetts General Hospital

**Keywords:** base excision repair, DNA repair, antibiotics, cell death, reactive oxygen species, ROS, essential gene

## Abstract

Various lethal stresses, including bactericidal antibiotics, can trigger the production of reactive oxygen species (ROS) that contribute to killing. Incomplete base excision repair (BER) of oxidized nucleotides, especially 8-oxo-dG, has been identified as a major component of ROS-induced lethality. However, the relative contributions of this pathway to death vary widely between stresses, due in part to poorly understood complex differences in the physiological changes caused by these stresses. To identify new lethal stresses that kill cells through this pathway, we screened an essential protein degradation library and found that depletion of either DapB or Dxr leads to cell death through incomplete BER; the contribution of this pathway to overall cell death is greater for DapB than for Dxr. Depletion of either protein generates oxidative stress, which increases incorporation of 8-oxo-dG into the genome. This oxidative stress is causally related to cell death, as plating on an antioxidant provided a protective effect. Moreover, incomplete BER was central to this cell death, as mutants lacking the key BER DNA glycosylases MutM and MutY were less susceptible, while overexpression of the nucleotide sanitizer MutT, which degrades 8-oxo-dGTP to prevent its incorporation, was protective. RNA sequencing of cells depleted of these proteins revealed widely different transcriptional responses to these stresses. Our discovery that oxidative stress-induced incomplete BER is highly dependent on the exact physiological changes that the cell experiences helps explain the past confusion that arose concerning the role of ROS in antibiotic lethality.

## INTRODUCTION

Bacterial cell death is a complex and poorly defined process. In addition to inhibition of an essential target or pathway, which can have direct lethal effects, potentially lethal stresses can have wide-reaching effects on a cell’s physiology as it attempts to survive, which in turn can lead to other forms of lethality (1). The production of reactive oxygen species (ROS) has been proposed as a common response to numerous lethal stresses, including multiple classes of bactericidal antibiotics, in the presence of which ROS causally contribute to the mechanism of killing (2–6). Although this idea was initially questioned (7, 8), further research on the mechanisms of death from bactericidal antibiotics (4, 9–15), as well as on other lethal stresses, such as type VI secretion systems (T6SS), P1*vir* phage, the antimicrobial peptide polymyxin B (16), MalE-LacZ_72–47_ (herein referred to as MalE-LacZ) expression (13), thymineless death (TLD) (11), and DnaB inactivation (15), has strongly supported the conclusion that endogenously produced ROS can contribute to bacterial cell death. The exact origin of this ROS is incompletely understood but is tightly connected to metabolic activity (17–19). Some ROS, such as hydrogen peroxide and superoxide, have a limited ability to directly damage cellular components (20, 21). However, hydrogen peroxide can react with Fe^2+^ through the Fenton reaction and produce hydroxyl radicals (**^·^**OH), which are highly reactive and capable of damaging a wide array of cell components (9, 20, 21). The nucleotide pool is particularly susceptible to this kind of damage, as nucleotide triphosphates are capable of complexing with Fe^2+^ (22, 23). Since the diffusion distance for a hydroxyl radical is only one carbon bond length (24), the localized production of Fenton oxidants favors the oxidation of the attached base.

Guanine is the most easily damaged nucleotide, with dGTP being readily oxidized to 8-oxo-dGTP. Incorporation of this nucleotide into the genome is highly mutagenic due to its ability to pair with dA as well as dC. Two components of the well-studied GO system (25) limit the consequences of 8-oxo-dG: the nucleotide sanitizer MutT, which degrades 8-oxo-dGTP to 8-oxo-dGMP to prevent its incorporation into the genome, and the base excision repair (BER) glycosylases MutM (Fpg) and MutY, which repair the genome itself (25). MutM directly removes 8-oxo-dG residues and leaves a single-strand break that is subsequently processed and filled, while MutY removes mispaired adenines across from 8-oxo-dG and leaves an apurinic site that requires processing by an apurinic/apyrimidinic (AP) endonuclease (26). The repair intermediates produced by these initial steps are DNA lesions themselves, which are potentially more dangerous than the original damage introduced by oxidative stress. A replication fork encountering a single-strand break can result in a double-strand break (DSB), as can the introduction of two or more single-strand breaks in close proximity (27). If left unrepaired, a DSB is lethal to the cell (28). This general pathway of incomplete BER-mediated cell death has been described for both bacteria and eukaryotes, where it can causally contribute to cell death (6, 29).

While incomplete BER has been identified as playing a role in cell death from a variety of unrelated stresses, its relative contributions to lethality vary widely. In the striking case of expression of a MalE-LacZ fusion protein, we have shown that incomplete BER is responsible for almost all cell death (13), whereas for bactericidal antibiotics, it is but one component of overall cell death, with the magnitude of its contribution varying substantially depending both on the specific antibiotic and the growth conditions experienced by the cells (3, 6).

To better understand how and when incomplete BER contributes to cell death, we were interested in identifying and characterizing new unrelated lethal stresses that involve this pathway. We screened an Escherichia coli essential protein degradation library in which a single essential protein can be degraded by an inducible protease (30) under various conditions that would protect against incomplete BER-mediated cell death. We identified two essential proteins not previously associated with this pathway, DapB and Dxr, and confirmed that depletion of either induces oxidative damage and that incomplete BER then contributes causally to cell death. While DapB depletion was found to closely mirror the consequences of MalE-LacZ expression, where incomplete BER was the dominant pathway of cell death, for Dxr, it was a minor contributing factor similar to that of some antibiotics. We also performed transcriptome analyses following depletion of these two proteins and observed highly disparate responses. Our results show that ROS production, oxidation of dG to 8-oxo-dG, and subsequent incomplete BER can occur as a result of very different stresses and that the relative contributions of incomplete BER to killing can also greatly differ depending on what other physiological changes a particular stress has induced.

## RESULTS

### Screening an essential protein degradation library to identify stresses that result in cell death due to incomplete BER.

In the E. coli essential protein degradation library constructed by Cameron and Collins (30), expression of the Mesoplasma florum Lon (*Mf*-Lon) protease is induced through the addition of anhydrotetracycline (ATc), resulting in the degradation of the tagged essential protein. This library contains 238 proteins essential for aerobic growth in LB Lennox medium, and depletion of any of these proteins results in either cell death or growth arrest.

These strains were screened for differential survival under various conditions that are known to affect the incomplete BER pathway of cell death. First, the essential proteins were depleted in the presence of the iron chelator bipyridyl, which limits the availability of iron for Fenton chemistry, thereby protecting DNA from oxidative damage (3). This experiment was performed with the cells exposed to bipyridyl during exponential growth in both liquid culture and on solid media containing the inducer. Second, they were plated on media and grown anaerobically to prevent the formation of ROS. Finally, each strain was transformed with a plasmid expressing the 8-oxo-dG sanitizer MutT, whose overexpression has previously been shown to be protective (3, 13). Each strain that registered as positive in more than one of these screens was retested individually.

This screening approach was internally validated by its identification of four proteins already known to be associated with incomplete BER-mediated cell death.

### (i) GyrA, a component of DNA gyrase (Fig. 1A).

The fluoroquinolone class of bactericidal antibiotics targets gyrase by trapping the enzyme in a DNA-protein complex. The generation of ROS and incomplete BER are well established to be contributing factors to the lethality of fluoroquinolone antibiotics (3, 31).

### (ii) FolC, part of the tetrahydrofolate biosynthesis pathway (Fig. 1B).

FolC is upstream of FolA, the direct target of the bactericidal antibiotic trimethoprim. Inhibiting this pathway blocks thymine synthesis, and trimethoprim treatment has many similarities with the phenomenon of thymineless death (TLD). Both have been shown to cause cell death at least partially through incomplete BER (5, 11).

### (iii) SecY, a component of the *sec*-dependent secretion pathway (Fig. 1C).

The expression of the historically important chimeric periplasmic-cytoplasmic MalE-LacZ_72–47_ fusion protein (herein referred to as MalE-LacZ) jams the *sec*-dependent secretion system (32), which results in cell death predominantly through incomplete BER (13).

### (iv) LexA, a regulator of the SOS response (Fig. 1D).

Degradation of LexA is known to trigger a strong SOS response, which includes the translesion DNA polymerase DinB. Overexpression of DinB has long been known to be lethal, and it was one of the earliest examples identified of a lethal stress that kills cells through incomplete BER-mediated cell death due to its high propensity to use 8-oxo-dGTP as a substrate (3).

Conversely, DnaB was not identified in our screen despite being present in the library. Previous work demonstrated that heat inactivation of a temperature-sensitive allele of the DnaB gene is another stress that induces cell death through the production of ROS (15). This is possibly a result of a difference in the kinetics, as protein function can be rapidly restored after heat inactivation. In contrast, the *Mf*-Lon protease might persist for a while even without the inducer, resulting in a longer period of protein depletion that may result in cell death through a different, more direct pathway.

**FIG 1:**
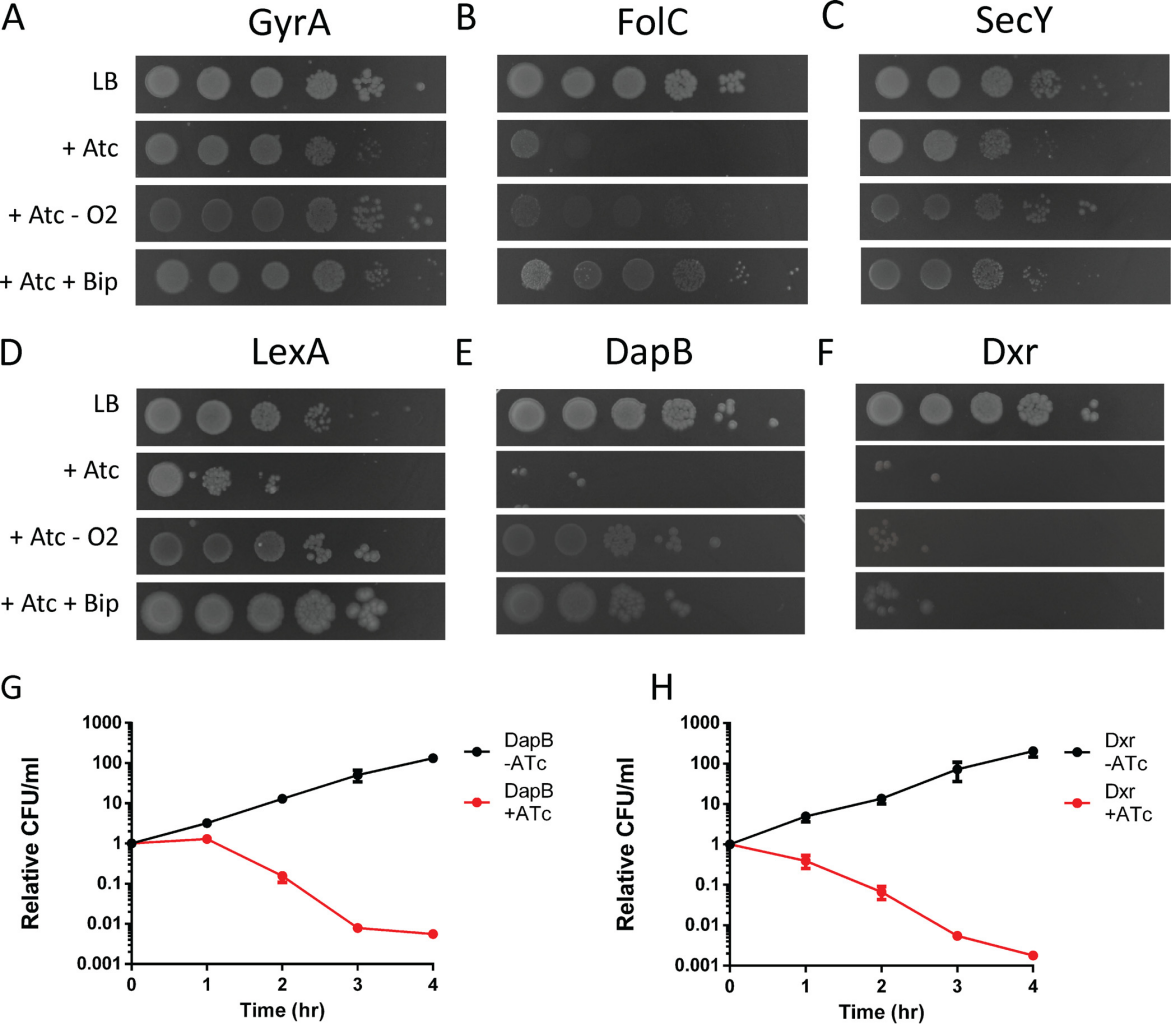
The lethality caused by depletion of certain essential proteins can be limited by bipyridyl (Bip; 250 μM) or anaerobic growth. (A) Several hits were in proteins already known to involve this pathway of cell death, including GyrA, the target of fluoroquinolone antibiotics. (B to D) FolC (B), upstream of the antibiotic trimethoprim SecY (C), a component of the secretion system blocked by MalE-LacZ LexA (D), an SOS regulator that represses DinB. (E and F) Two new proteins were also identified, DapB (E), an enzyme in the DAP production pathway, and Dxr (F), an enzyme in the MEP pathway of isoprenoid biosynthesis. (G and H) Depletion of DapB (G) and Dxr (H) cause a 100-fold loss in the number of CFU over 4 h; data are means ± standard deviations (SD) from 3 biological replicates.

Our screen resulted in the identification of two proteins not previously associated with the incomplete BER pathway of cell death, DapB and Dxr (Fig. 1E and F). The first of these, DapB, is 4-hydroxy-tetrahydrodipicolinate reductase, an enzyme in the lysine biosynthetic pathway (33). Since lysine is present in the medium in which the cells are grown, its biosynthesis is not essential. However, this pathway also produces the amino acid diaminopimelic acid (DAP), which is used to form the peptide cross-link in peptidoglycan in many bacteria. Since humans lack this pathway, it has been considered a promising antibiotic target (34). The second protein, Dxr, is 1-deoxy-d-xylulose 5-phosphate reductoisomerase, which catalyzes the first committed step in the methylerythritol phosphate (MEP), or nonmevalonate, pathway of isoprenoid biosynthesis (35). Isoprenoids include a wide array of important biological molecules, including the quinones, which act as electron transport carriers, and the undecaprenol lipid carrier required for cell wall and outer membrane biosynthesis (36). Since mammals use the mevalonate pathway to synthesize isoprenoids, the nonmevalonate pathway has been a major focus of antibiotic development since it is used by most bacteria (37). Additionally, apicomplexan parasites, such as *Plasmodium*, also rely on this pathway (38), which makes it an attractive target for new antimalarials. The antibiotic fosmidomycin targets both Dxr (39) and the next enzyme in the same pathway, IspD (40), and has been shown to be a promising antimalarial in clinical trials (41).

### DapB or Dxr depletion generates ROS that contribute to cell death.

**FIG 2:**
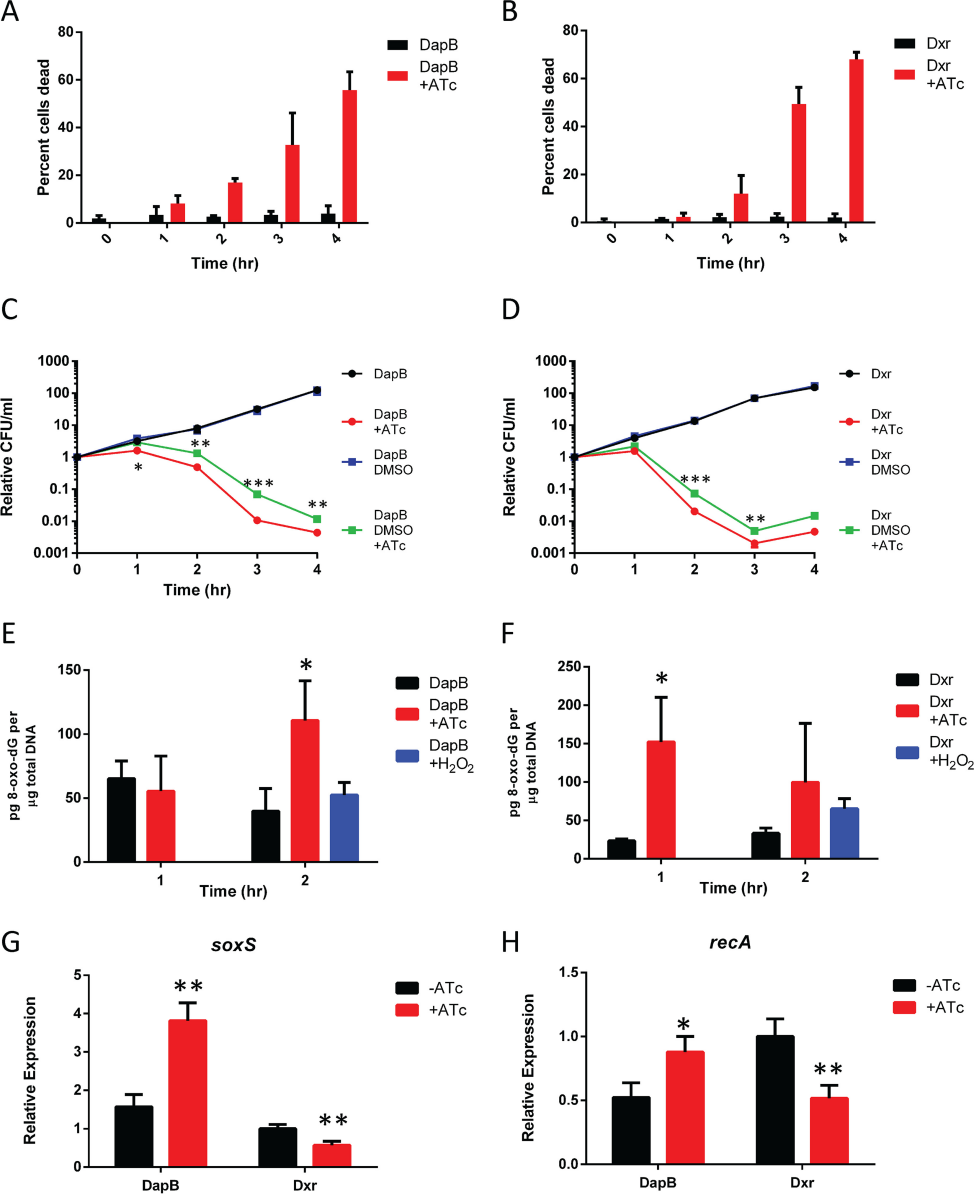
Depletion of DapB and Dxr leads to ROS-mediated cell death. (A and B) Quantification of live-dead staining shows that depletion of both DapB (A) and Dxr (B) leads to a loss of membrane potential, which lags behind the loss of CFU. (C) Plating DapB-depleted cells on agar containing DMSO, which limits the activity of ROS, leads to a significant reduction in lethality. (D) Plating on DMSO-containing plates provides a more modest protective effect for Dxr depletion. (E and F) DapB (E) and Dxr (F) depletion leads to an increase in the 8-oxo-dG content of the genome as measured by an ELISA, with 10 mM H_2_O_2_ serving as a positive control. (G) DapB depletion leads to an increase in *soxS* expression, while Dxr depletion leads to a decrease, as determined by qPCR. (H) RecA expression is increased by DapB depletion and decreased by Dxr depletion. Data are means ± SD from 3 biological replicates. Each data set is a representative result from at least three independent experiments. *, *P* < 0.05; **, *P* < 0.01; ***, *P* < 0.001; using Student's *t* test.

Depletion of either DapB or Dxr results in a 2-log loss of cell viability within 4 h (Fig. 1G and H). As expected, the addition of diaminopimelic acid to the medium allows for cell growth even under DapB depletion conditions (see Fig. S1 in the supplemental material). Interestingly most of these cells will fail to form colonies when plated on solid media, and this loss of viability can be prevented by plating on LB containing DAP (Fig. S2). This is likely due to a combination of lingering *Mf*-Lon protease and the time that it takes to synthesize enough DapB, supporting our hypothesis for why we failed to identify DnaB in our screen. Light microscopy of cells at various time points showed no significant change in cell morphology, and in both cases, cell death was largely nonlytic (Fig. S2A and B). While numbers of CFU dropped markedly 2 h after the induction of the protease for both DapB and Dxr (Fig. 1G and H), it took until the 3-h or 4-h time point for a majority of cells to die by the criterion of live-dead staining (Fig. 2A and B). This pattern of lagging death has been reported previously after expression of the MalE-LacZ fusion protein (13) and in other instances where oxidative stress is a contributing factor to cell death (15). These stresses are capable of committing the cell to death since even when the lethal stress is removed, the resulting oxidative stress can persist and continue to damage and potentially kill the cell (15). Mi et al. have shown that this can be mitigated by plating the bacteria on media containing an antioxidant, such as dimethyl sulfoxide (DMSO) (12). Plating either DapB- or Dxr-depleted cells onto medium containing DMSO provides a modest protective effect (Fig. 2C and D), with it being much more effective at preventing cell death from DapB depletion.

10.1128/mbio.03756-21.1 FIG S1 (A) The addition a 0.5 mM concentration of the amino acid DAP to the media allows cells to grow under DapB depletion conditions, albeit at a reduced rate. Lower concentrations result in a dramatically decreased ability of the cells to grow. (B) Plating DapB-depleted cells grown with 0.5 mM DAP on solid LB agar with DAP results in a substantial decrease in the number of CFU. The vitality of these cells can be maintained by plating them on LB agar with 0.5 mM DAP. Growth curve data are means from 6 biological replicates. Kill curve data are means ± SD from 3 biological replicates. Download FIG S1, TIF file, 0.07 MB. Copyright © 2022 Gruber et al. 2022 Gruber et al. https://creativecommons.org/licenses/by/4.0/ This content is distributed under the terms of the Creative Commons Attribution 4.0 International license.

10.1128/mbio.03756-21.2 FIG S2 DapB and Dxr depletion leads to cell death. (A and B) Live-dead staining shows the loss of cell viability after DapB depletion (A), with around half the cells dead after 4 h, while Dxr depletion causes cell death slightly faster, with a majority dead by the 3-h time point (B). Download FIG S2, TIF file, 0.3 MB. Copyright © 2022 Gruber et al. 2022 Gruber et al. https://creativecommons.org/licenses/by/4.0/ This content is distributed under the terms of the Creative Commons Attribution 4.0 International license.

Consistently with their depletion leading to ROS generation, we found that degradation of either DapB or Dxr leads to an increase in the genomic content of 8-oxo-dG (Fig. 2E and F). 8-oxo-dG is not only a well-established marker of oxidative stress but a requirement for the model of incomplete BER-mediated cell death that we have proposed (3, 6, 13). As with other lethal stresses, such as MalE-LacZ expression, the T6SS, and P1*vir* phage (13, 16), DapB depletion increases the expression of the oxidative-stress regulator *soxS* (Fig. 2G). In contrast, Dxr depletion actually lowers the expression of *soxS* (Fig. 2G), suggesting that despite some commonalities, there are significant differences in cells’ responses to these two lethal stresses. Similarly, DapB depletion slightly increased the expression of the DNA repair gene *recA*, while Dxr depletion decreased it (Fig. 2H).

### Incomplete BER is an important cause of cell death for both DapB and Dxr depletion.

**FIG 3:**
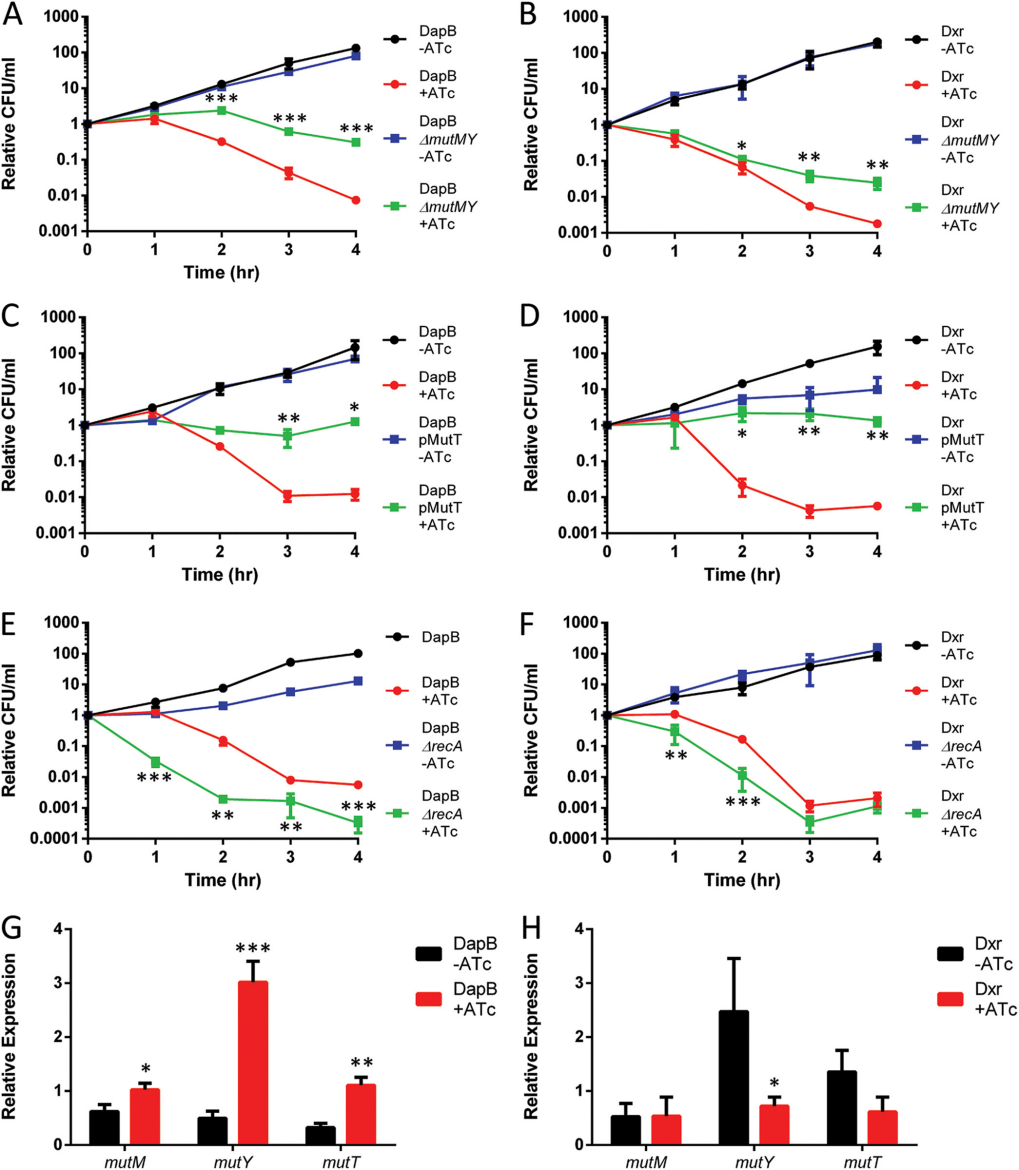
BER of 8-oxo-dG causally contributes to cell death from DapB and Dxr depletion. (A) A *mutM mutY* glycosylase double mutant is much more resistant to death from DapB depletion. (B) The *mutM mutY* double mutant is also more resistant to Dxr depletion. (C and D) Overexpression of the 8-oxo-dGTP sanitizer MutT negates the bactericidal effects of DapB (C) and Dxr (D) depletion. (E and F) A knockout of the DNA repair gene *recA* is sensitized to DapB (E) and Dxr (F) depletion. (G) DapB depletion increases the expression of *mutM*, *mutY*, and *mutT* as measured by qPCR. (H) Dxr depletion decreases the expression of *mutY*. Data are means ± SD from 3 biological replicates. Each data set is a representative result from at least three independent experiments. *, *P* < 0.05; **, *P* < 0.01; ***, *P* < 0.001; using Student's *t* test.

One important element of our model for incomplete BER-mediated cell death in response to oxidative stress is that a significant source of the increased 8-oxo-dG in the genome is from the use of 8-oxo-dGTP by DNA polymerases. Overexpression of the 8-oxo-dGTP sanitizer MutT reduces killing from various lethal stresses that have been associated with incomplete BER (3, 6, 13), and we found that MutT overexpression provides a protective effect against depletion of DapB and Dxr (Fig. 3C and D).

A second important element of our model of incomplete BER-mediated cell death is that the BER DNA glycosylases MutM and MutY play causal roles in cell death (3, 6), since knocking out both the *mutM* and *mutY* genes has been protective for many different lethal stresses (3, 5, 11, 13, 42). Double mutations were constructed in both the DapB and Dxr depletion strains, and cell death was measured after induction of the protease. Cell death from depletion of DapB was largely prevented in the *mutM* and *mutY* double mutant (Fig. 3A), while death from the depletion of Dxr was reduced (Fig. 3B). These observations suggest that while oxidative stress and incomplete BER play a role in cell death in both cases, it is a larger component of the death from DapB depletion. This is in agreement with the larger protective effect of DMSO for DapB depletion.

After the actions of BER glycosylases, the resulting DNA problems that threaten the cell may potentially be repaired in a fashion that requires RecA, which is essential for many repair pathways, including DSB and single-strand gap repair (43). As expected by our model, both of the depletion strains with *recA* knocked out were significantly more susceptible to killing (Fig. 3E and F). Interestingly, the Δ*recA* mutants also lost the brief lag period before cell death begins that is normally present after the induction of the protease. This is extremely similar to the pattern seen with MalE-LacZ expression (13). This suggests that the damage arising from DapB and Dxr depletion begins quite early after the induction of the protease but is initially kept in check through RecA-mediated DNA repair before being overwhelmed.

### Imbalances in the ratios of BER components can favor cell death due to incomplete BER.

Since the various intermediates in BER can be more toxic that the original lesions, the various stages of BER must be tightly coordinated, and this coordination was originally compared to the passing of a baton, where the repair product of each enzyme in the BER pathway is handed over to the next enzyme (44). A consequence of this key principle of BER is that imbalances between the various components of the repair process can result in incomplete BER and thus result in a situation where the repair intermediates can prove toxic.

We have previously observed that cell death due to the expression of the MalE-LacZ fusion protein greatly increased the expression of MutM and hypothesized that this imbalance contributes to incomplete BER being its primary pathway of cell death (13). In a conceptually similar fashion, we observed that depletion of DapB greatly increased the expression of MutY, with smaller increases in MutM and MutT (Fig. 3G). In contrast, depletion of Dxr leads to a slight decrease in the expression of MutY (Fig. 3H). These imbalances in the levels of these BER proteins caused by these stresses may be an important factor in why incomplete BER contributes to cell death from DapB and Dxr depletion.

### Transcriptomics of DapB and Dxr depletion.

**FIG 4:**
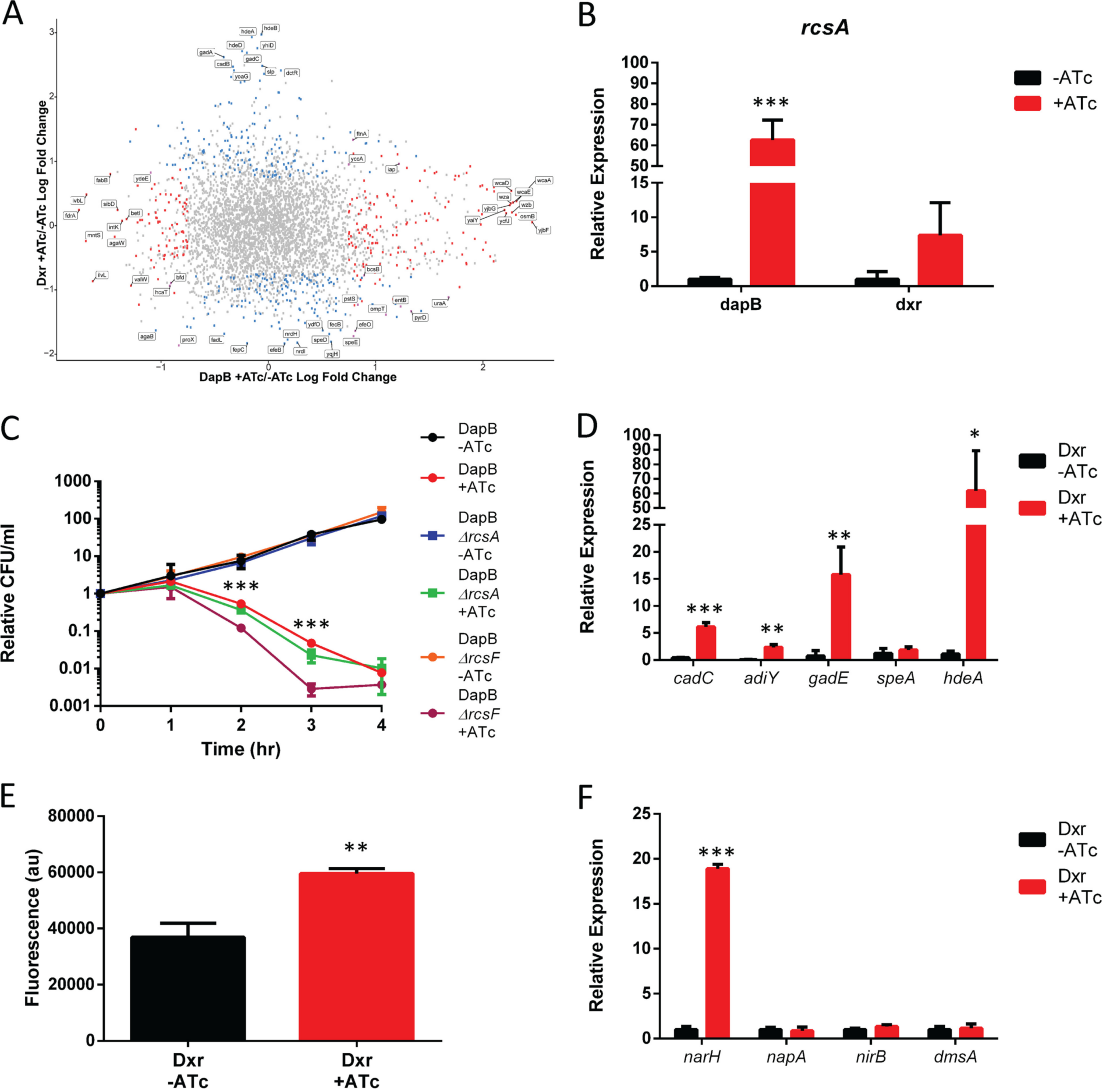
DapB and Dxr depletion cause extremely different transcriptional responses despite incomplete BER contributing to cell death in both. (A) RNA sequencing was performed on both DapB- and Dxr-depleted cells. Genes are positioned on the *x* axis based on their log fold change in gene expression following DapB depletion and on the *y* axis following Dxr depletion. Red dots were genes affected only by DapB depletion, blue dots were genes affected by Dxr depletion, and purple dots were genes affected by both. There was almost no overlap in changes to gene expression. (B) DapB depletion causes an extremely strong activation of the *rcs* regulon through increased expression of the regulator RcsA. Dxr depletion also increases RcsA expression. (C) An *rcsF* knockout, but not an *rcsA* knockout, sensitizes cells to DapB depletion, similar to what has been reported for β-lactam antibiotics. (D) Dxr depletion results in a strong activation of acid stress systems, including three of the four amino acid antiporter systems, namely, LDAR (*cadC*) ADAR (*adiY*), and GDAR (*gadE*), as well as the acid stress gene *hdeA*, but not the ODAR system (*speF*). (E) Dxr depletion results in a decrease in cytoplasmic pH as measured by the increase in the fluorescence of a pH-sensitive dye. (F) Dxr depletion greatly increases the expression of the cytoplasmic nitrate reductase system (*narH*). Data are means ± SD from 3 biological replicates. Each data set is a representative result from at least three independent experiments. *, *P* < 0.05; **, *P* < 0.01; ***, *P* < 0.001; using Student's *t* test.

DapB and Dxr are in completely unrelated metabolic pathways, and their depletion is thus expected to result in widely different physiological consequences. Despite this, in both cases, cell death is partially due to incomplete BER of DNA lesions associated with 8-oxo-dG. To gain insights into whether there are any similarities in the cells’ responses to these lethal stresses, we performed RNA sequencing (RNA-Seq) at several different times points after induction of the *Mf*-Lon protease and compared the changes in transcription. The results of DapB and Dxr depletion were extremely distinct, with few similarities (Fig. 4A).

The depletion of DapB very strongly activates the Rcs regulon (Fig. 4B). This is also a feature of β-lactam antibiotics, which is not unexpected since depletion of DapB also results in an inability to cross-link peptidoglycan. In the case of β-lactam antibiotics, the Rcs phosphorelay system senses perturbations in the outer membrane through the outer membrane lipoprotein RcsF. This eventually results in the phosphorylation of the transcription factor RcsB, which then activates a potentially protective response (45). In contrast, RcsA, which is responsible for the regulation of capsule synthesis, is not required for this protective response (46). Knocking out *rcsF* and *rcsA* in the DapB depletion strain resulted a pattern similar to that for β-lactam antibiotics, where RcsF is protective but RcsA is not (Fig. 4C).

Depletion of Dxr also modestly activates the Rcs regulon (Fig. 4B), but it very strongly upregulates several acid stress systems (Fig. 4C). Three of E. coli’s four acid stress-induced amino acid antiporter systems were upregulated, LDAR (*cadC*), ADAR (*adiY*), and GDAR (*gadE*) but not ODAR (*speA*), as well as other acid stress genes, such as *hdeA* (Fig. 4D). In contrast, the expression of the Cpx regulon, which is implicated in sensing periplasmic pH, was unchanged (Table S1) (47). These observations suggested a drop in cytoplasmic pH, which was confirmed using a pH-sensitive fluorescent dye (Fig. 4E).

10.1128/mbio.03756-21.3 TABLE S1 Log fold changes in expression of E. coli genes due to depletion of DapB or Dxr 2 and 3 h after the induction of the *Mf*-Lon protease. Download Table S1, XLSX file, 0.6 MB. Copyright © 2022 Gruber et al. 2022 Gruber et al. https://creativecommons.org/licenses/by/4.0/ This content is distributed under the terms of the Creative Commons Attribution 4.0 International license.

Dxr depletion also upregulated genes involved with anaerobic respiration. Nitrate reductase and the genes responsible for producing their various cofactors were all strongly expressed. The Nar system is E. coli’s preferred anaerobic respiration system under high-nitrate conditions, such as those that are found in LB (48), and was the only system that was upregulated (Fig. 4F). Expression of all of these genes was expected to be strongly repressed under the aerobic conditions in which the cells were grown.

**FIG 5:**
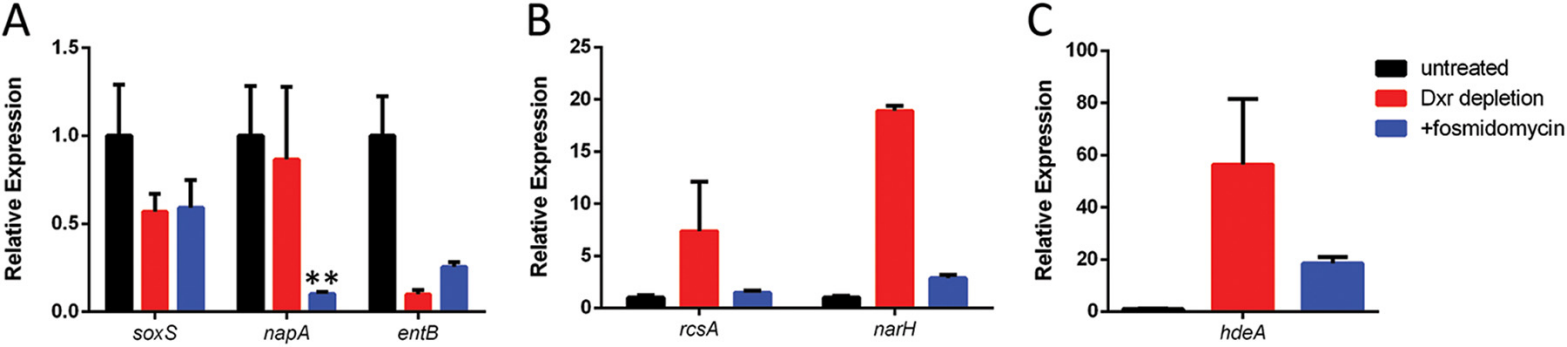
Fosmidomycin treatment mirrors Dxr depletion. (A) Both drug treatment and Dxr depletion lower the expression of *soxS* and *entB*, while fosmidomycin also lowers the expression of *napA*, a subunit of the periplasmic nitrate reductase. (B) Both treatments increase the expression of the anaerobic respiration gene *narH*, but only *dapB* depletion increases the expression of *rcsA*. (C) Expression of the acid stress gene *hdeA* is increased by both treatments, although it is expressed significantly more with Dxr depletion. Data are means ± SD from 3 biological replicates. Each data set is a representative result from at least three independent experiments. *, *P* < 0.05; **, *P* < 0.01; ***, *P* < 0.001; using Student's *t* test.

Having identified several major markers of stress caused by Dxr depletion, we examined whether the degradation of this protein recapitulates the transcriptional response that occurs as a result of Dxr inhibition by the antibiotic fosmidomycin. As expected, the levels of expression of *soxS*, *entB*, *rcsA*, *hdeA*, and *narH* were affected somewhat similarly between Dxr depletion and fosmidomycin treatment, although the magnitudes of change varied greatly (Fig. 5A and B), suggesting that protein depletion roughly mimics antibiotic activity.

**FIG 6:**
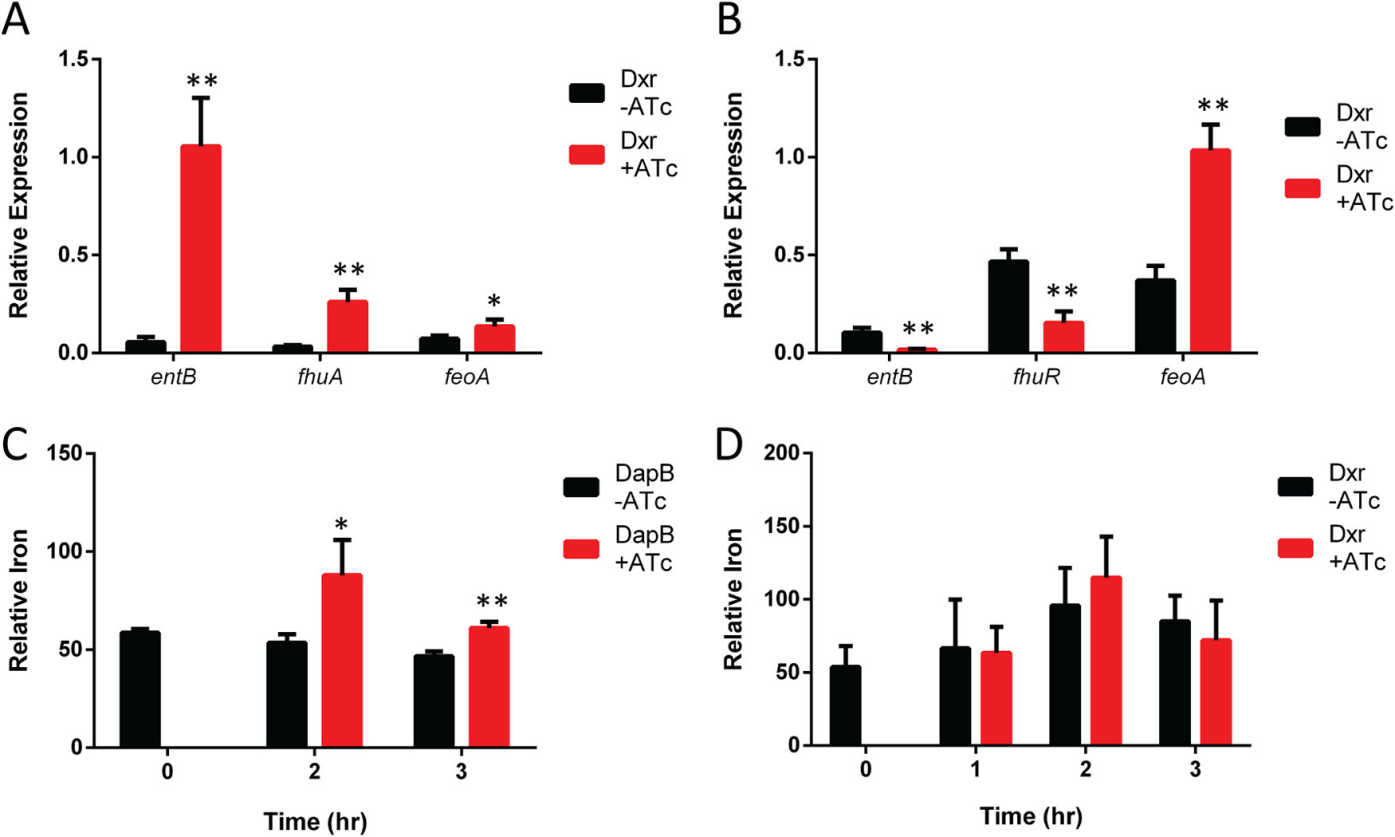
Both DapB depletion and Dxr depletion alter the expression of iron uptake genes and lead to increased levels of iron in DapB-depleted cells. (A) DapB depletion causes an increase in the expression of three different iron uptake systems, enterobactin (*entB*), ferrichrome (*fhuA*), and ferrous iron (*feoA*). (B) Dxr depletion causes a decrease in enterobactin (*entB*) and ferrichrome (*fhuA*) but an increase in ferrous iron (*feoA*). (C and D) DapB depletion leads to an elevation in cellular iron levels (C), while the amount of iron in Dxr-depleted cells is unchanged (D). Data are means ± SD from 3 biological replicates. Each data set is a representative result from at least three independent experiments. *, *P* < 0.05; **, *P* < 0.01; ***, *P* < 0.001; using Student's *t* test.

Interestingly, one of the few genes whose expression changes were divergent between DapB and Dxr depletion (Fig. 4, lower right quadrant) was the iron uptake gene *entB.* DapB depletion increases the expression of iron uptake systems, such as enterobactin (*entB*), ferrichrome (*fhuA*), and ferrous iron (*feoA*) (Fig. 6A). In contrast, Dxr depletion resulted in a drop in the expression of both the enterobactin and ferrichrome transport systems, while the expression of the ferrous iron transport system increased (Fig. 6B). The amount of iron in cells was measured using inductively coupled plasma mass spectrometry (ICPMS), and DapB depletion resulted in elevated iron levels (Fig. 6C). Despite the transcriptional changes of the iron uptake systems, the amount of iron in Dxr-depleted cells was unchanged (Fig. 6D).

## DISCUSSION

ROS and incomplete BER have been found to play a role in cell death from a variety of lethal stresses. We identified two new stresses that also follow this pattern: the depletion of the essential proteins DapB and Dxr. Despite these two stressors sharing a component of lethality, we found their physiological responses to be extremely different. This is consistent with previous conclusions that there is not a singular pathway that leads to ROS production but rather that it is a common metabolic consequence of greatly perturbed cellular physiology. For example, several different metabolically distinct futile cycles all lead to increased ROS production (17). Additionally, the relative contributions of incomplete BER to cell death differed between the two stressors, supporting our hypothesis that susceptibility to this pathway of cell death is heavily dependent on the exact physiological changes that caused the lethal stress, with respect to both the amount of ROS produced and the degree of perturbation of the cell machinery involved in 8-oxo-dG-related DNA repair (13).

The depletion of DapB has several features that mirror those of cell wall synthesis inhibitors, such as β-lactams. This was expected, as DapB is required to produce the DAP that Gram-negative bacteria, such as E. coli, use to cross-link peptidoglycan. The transcriptional changes and effects of *rcsA* and *rcsF* mutations parallel those of β-lactam treatment. Interestingly, the relative lack of cell lysis suggests a greater similarity to β-lactams that inhibit class B penicillin biding proteins (PBP) (49) than to those that inhibit class A PBPs, which generally result in cell lysis (50). Unlike with small-molecule inhibitors that show different specificities for various PBPs, DapB depletion should have a more unbiased effect. Additionally, the lack of DAP for cross-linking may trigger the same futile energy-intensive cycle of cell wall synthesis and degradation that β-lactam antibiotics are capable of triggering (51).

DapB depletion also resulted in a number of characteristics shared with previously described lethal stresses. As with MalE-LacZ expression, the type VI secretion system, and P1*vir* phage, expression of the oxidative-stress regulator SoxS was increased (13, 16), and like MalE-LacZ, the OxyR regulon was unaffected. The level of ROS induced by these stresses is quite low (4), potentially below the threshold required for activation of the OxyR response, which would limit the protective response against oxidative damage. DapB depletion also increases the expression of certain 8-oxo-dG repair proteins, particularly MutY, while MalE-LacZ expression increases the expression of MutM. Increased expression of these glycosylases without a corresponding increase in downstream BER components would result in an increase in BER intermediates in the genome, thus increasing the risk of one of these lesions being encountered by a replication fork and causing a DSB. Interestingly, MutY is normally downregulated by oxidative stress (52), while MutM is regulated by the heat shock response (53). This suggests that E. coli evolved to not upregulate these genes under oxidative-stress conditions as a strategy to limit the accumulation of these potentially dangerous DNA repair intermediates.

In contrast, Dxr depletion had little in common with any previously described stressors, with no activation of SoxS or downregulation of MutY, which may explain the lower level of contribution of incomplete BER to overall cell death. The transcriptional changes that were identified suggest a greatly altered cell physiology. Of particular interest was the strong induction of anaerobic respiration, as these genes should be repressed under aerobic conditions. One of our predicted outcomes of Dxr depletion was the inability of the cell to produce new quinones due to the block in isoprenoid biosynthesis. The histidine kinase of the ArcAB two-component system uses oxidized quinones as a signal that the cell is growing aerobically (54). This system has significant overlap and interplay without other regulators, such as FNR (55, 56), so this may explain why Dxr-depleted cells express anaerobic respiration systems. Another major change in physiology was the acidification of the cytoplasm. Previous work has found that the *marRAB* operon is responsible for the slight acidification of E. coli’s cytoplasm (pH ∼7.4 to 7.7 → ∼7.1 to 7.5) in response to the antibiotic norfloxacin, and this helps facilitate resistance (57). However, while expression of *marRAB* is increased by Dxr depletion (Table S1), the massively elevated transcription of multiple acid stress systems suggests a much lower pH of ∼6.5 (58). The exact causes and consequences of this change in pH still need to be elucidated.

One of the very few similarities between DapB and Dxr depletion was that both affected iron uptake, albeit largely in different directions. Iron is central to Fenton chemistry and the generation of hydroxide radicals that are required to generate 8-oxo-dG, so the levels of cellular iron may have profound effects on incomplete BER-mediated cell death. The increase in uptake and cellular iron levels in DapB-depleted cells would put them at higher risk for Fenton chemistry-generated radicals. Dxr depletion on the other hand was able to maintain normal iron levels in spite of several uptake systems being downregulated. The one upregulated system, the ferrous iron transport system, in E. coli is regulated by the ArcAB system and is the preferred transporter under anaerobic conditions. Cellular physiology can also affect the rate of Fenton chemistry, particularly pH (59). The Fenton reaction is promoted by higher pH, so the protective effective of *marRAB*-induced acidification of the cytoplasm observed by Reyes-Fernandez and Schuldiner (57) may be in part due to suppressed Fenton chemistry, which may also contribute to the reduced role of the BER-mediated pathway of cell death in Dxr depletion.

The vastly different physiological responses to these two lethal stresses highlight the importance of altered cellular homeostasis to incomplete BER-mediated cell death, as it is unlikely that endogenously generated ROS alone is sufficient to cause cell death (60). For DapB depletion, increased Fenton chemistry due to a higher availability of iron and a higher rate of BER incisions is the likely candidate for the observed effects. Conversely, what physiological changes Dxr depletion causes that allow for ROS and incomplete BER to contribute to cell death are still unknown and will require further research. In addition to Dxr’s differing effects on cell physiology, timing may play an important role in the various levels of incomplete BER-mediated cell death. Due to the protective effects of MutT overexpression and MutY knockouts, at least one round of DNA replication is required in our model. In all experiments, the Dxr-depleted cells died earlier than the DapB-depleted cells, suggesting that Dxr depletion is more immediately toxic to the cells. There may simply be less time for 8-oxo-dG lesions to accumulate before the cell dies due to another cause.

In the end, there is a finite number of ways for a bacterial cell to die, and a lethally stressed cell may experience multiple pathways of cell death at the same time. The exact “causes of death” can vary from cell to cell, and the relative levels of each cause of death vary from condition to condition. These complexities of cell death likely confounded early research into the role that oxidative stress plays in bacterial cell death from antibiotic treatment (2, 3, 7, 8). In retrospect, we note that key publications chiefly responsible for casting doubt on the role that ROS play in antibiotic lethality (7, 8) neglected previously published evidence (3) for 8-oxo-dG-dependent incomplete BER being an underlying causal mechanism for the oxidative component of antibiotic lethality. This was an important omission, since a single DNA DSB is sufficient to kill a cell and can result from a very modest increase of 8-oxo-dG in the genome, coupled with a physiologically induced imbalance in BER enzymes, as described above. Instead, an argument was made that it is not plausible for antibiotics to induce enough ROS to kill cells by processes that require bulk action, such as inactivating mononuclear iron enzymes or iron-sulfur dehydratases (60). It also led to the conclusion that Fenton chemistry was not likely to be important at the DNA level, an inference that did not allow for the possibility that antibiotic-induced alterations of DNA repair proteins might create a vulnerability to oxidative killing by this mechanism.

Since then, a robust body of work has expanded on the number of stresses found be able to kill cells through oxidative stress from the initial three classes of bactericidal antibiotics, β-lactams, aminoglycosides, and fluoroquinolones (3, 4), to include the T6SS, the P1*vir* phage, polymyxin B (16), the MalE-LacZ fusion protein (13), thymineless death (11), and trimethoprim (5). Our research has specifically identified the incomplete removal of oxidized nucleotides as a major component of cell death (3, 4, 13). This study adds to the evidence that incomplete BER of oxidative lesions is a common pathway of cell death for a variety of stresses beyond those caused by antibiotics. The ubiquity of this pathway suggests that it might be a target for developing new antibiotics or increasing the efficacy of existing ones (3, 4, 13).

## MATERIALS AND METHODS

### Strains and plasmids.

All strains used in this study are listed in Table S1 in the supplemental material. Knockout mutants were created by phage P1 transduction of alleles from the Keio collection. Antibiotic cassettes were resolved using pEC201 (30). The MutT plasmid was from the ASKA plasmid collection (61).

### EPD library screen.

The Eukaryotic Promoter Database (EPD) strain library was screened multiple ways. First, cultures of each strain were grown overnight at 30°C and then diluted 1:100 in fresh LB Lennox and grown for 30 min at 30°C in a rotating shaker. The expression of the protease was induced through adding 50 ng/ml of anhydrotetracycline (Sigma). Five hundred micromolar bipyridyl or 100 mM thiourea was added to replicates, and samples were taken after 2 h, serially diluted, and spotted onto plates, which were then incubated overnight at 30°C. In a separate experiment, overnight cultures were serially diluted and spotted on LB plates containing 50 ng/ml ATc and 250 μM bipyridyl or 50 mM thiourea and incubated overnight at 30°C. For the anaerobic screen, overnight cultures were also serially diluted as before and spotted on LB with or without ATc. Plates were put into anaerobic canisters with a GasPak (BD) and incubated at 30°C for 48 h. To screen for MutT overexpression, each strain was transformed with the MutT plasmid or an empty vector control. Overnight cultures were diluted and grown for 30 min, and then 50 ng/ml ATc was added to induce the protease as before. Again, samples were taken after 3 h, and serial dilutes were spotted on LB agar and then incubated overnight at 30°C. Strains were considered hits if they were protected from killing in at least three of these six assays.

### EPD killing curve.

Each strain was streaked onto LB agar and grown overnight at 30°C. Three separate colonies were picked and grown overnight at 30°C in a rotating shaker. Cultures were diluted 1:100 in fresh LB and grown to early log phase, with an optical density at 600 nm (OD_600_) of ∼0.4, and diluted to an OD_600_ of 0.01; then 50 ng/ml of ATc was added. Samples were taken every hour, diluted, and plated on LB agar and then grown overnight at 30°C. For the antioxidant plates, the dilutes were plated on LB with 5% DMSO as well. Colonies were counted with a ProtoCOL 3 colony counter. Student's *t* test was used to determine if the difference between samples was statistically significant.

### Live/Dead assay.

The Live/Dead BacLight bacterial viability kit (ThermoFisher) was used to determine bacterial viability. The cultures were grown, and the degradation of essential protein was induced as previously described. Cells were collected at each time point and stained according to the manufacturer’s instructions. Cells were then plated on slide-mounted agarose pads on a Nikon Eclipse Ni microscope. Images were analyzed using ImageJ (62), and the numbers of live and dead cells were determined for each time point.

### 8-oxo-dG ELISA.

Cultures were grown and treated with ATc as described previously, and positive controls were treated with 10 mM H_2_O_2_ for 1 h. DNA was extracted using the DNA minikit (Qiagen) according to the manufacturer’s instructions. 8-oxo-dG was quantified using the HT 8-oxo-dG enzyme-linked immunosorbent assay (ELISA) kit II (Trevigen). Samples were assayed in biological triplicate.

### RNA sequencing and qPCR.

Cultures were initially prepared as before, except they were diluted to an OD_600_ of 0.1 when the ATc was added. Aliquots were taken at each time point, and RNA was extracted using the RNeasy bacterial minikit (Qiagen) and treated with the RNase-Free DNase set according to the manufacturers’ instructions. Single-end RNA-Seq reads were used to quantify gene expression by aligning against the E. coli MG1655 (ASM584v2) genome with Salmon (version 1.1.0) (63). The resulting counts and transcript per million (TPM) counts were assembled using the tximport package (version 1.12.0) (64). TPM counts were transformed to log_2_ space with an offset of 1. We imported the summarized integer counts into DESeq2 (version 1.24.0) (65, 66) and conducted differential analysis with apeglm log fold change shrinkage (67). As biological replicates were not available, all of the samples were treated as replicates of a single group for estimation of dispersion. We filtered the results that had a *P* value less than or equal to 0.05 and a log fold change greater than 1 or less than −1 to identify upregulated and downregulated genes, respectively. We identify enriched pathways by using DAVID Bioinformatics Resources (version 6.8) (68, 69) to identify functionally enriched pathways by comparing the lists of up-/downregulated genes against the background set. For quantitative PCR (qPCR), cDNA was created using an iScript cDNA synthesis kit (Bio-Rad) and was then used with PowerUp SYBR green (Life Technologies). For all DapB experiments, the 16S ribosomal gene *rrsA* and the *rpoA* subunit of the RNA polymerase were used as endogenous controls. For Dxr, *cysG* and *idnT* were used instead, as the *rrsA* and *rpoA* gene expression levels varied. All primers used are in Table S3.

10.1128/mbio.03756-21.4 TABLE S2 Strains used in this study. Download Table S2, DOCX file, 0.02 MB. Copyright © 2022 Gruber et al. 2022 Gruber et al. https://creativecommons.org/licenses/by/4.0/ This content is distributed under the terms of the Creative Commons Attribution 4.0 International license.

10.1128/mbio.03756-21.5 TABLE S3 Primers used in this study. Download Table S3, DOCX file, 0.01 MB. Copyright © 2022 Gruber et al. 2022 Gruber et al. https://creativecommons.org/licenses/by/4.0/ This content is distributed under the terms of the Creative Commons Attribution 4.0 International license.

### Fosmidomycin treatment.

For the fosmidomycin experiments, an inhibitory concentration of 50 μg/ml of fosmidomycin (70) was used in place of ATc. Cells were collected after 3 h of treatment and compared to cells with 3 h of Dxr depletion.

### Intracellular pH measurement.

Cells were grown and treated with ATc as previously described. Two hours after the induction of *Mf*-Lon, the cells were pelleted and washed with HEPES, pH 7.4, and stained with pHrodo green AM (Invitrogen) as per the manufacturer’s instructions. Fluorescence measurements where then performed using a Tecan Spark microplate reader.

### ICPMS measurements.

Metal content in cells was determined as previously described (71), with modifications. Samples were taken at various time points and were spun down and washed with metal-free water. The pellets were then digested in 40 μl 100% HNO_3_ and heated at 95°C for 1 h. Digested samples were diluted with 1,960 μl metal-free water (final concentration, 2% HNO_3_). The metal contents of the samples were determined in He mode using Agilent 7900 ICP-MS instrumentation. Similarly, a metal standard consisting of the divalent ions Mg^2+^, Mn^2+^, Ni^2+^, Fe^2+^, Co^2+^, and Zn^2+^ (1 mM each) was serially diluted, and metal counts were analyzed and plotted against concentration as standard curves. The metal counts of the samples were then calculated by fitting the metal counts with the standard curve for each metal.
