# Supplementary figures and images for: Degradation of the Escherichia coli Essential Proteins DapB and Dxr Results in Oxidative Stress, which Contributes to Lethality through Incomplete Base Excision Repair
Source: mBio. 2022 Feb 8;13(1):e03756-21. doi: 10.1128/mbio.03756-21 (PMC8822343; doi:10.1128/mbio.03756-21)

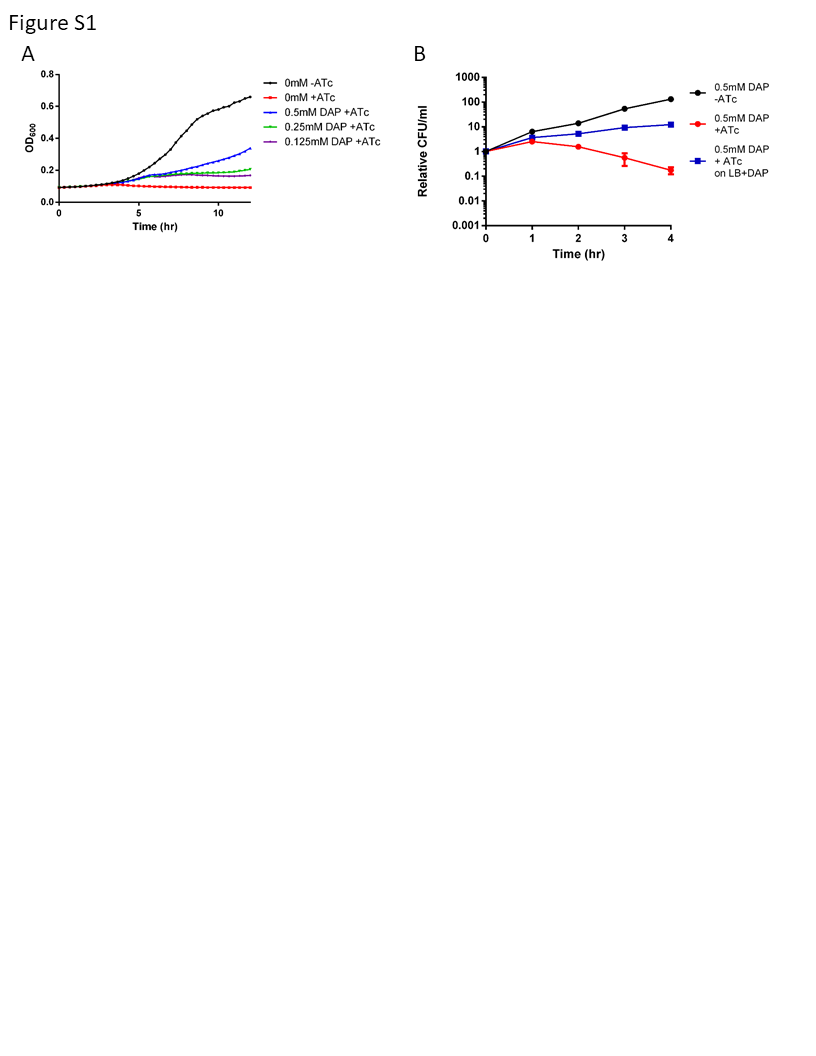

Supplement: FIG S1 [file mbio.03756-21-sf001.tif]

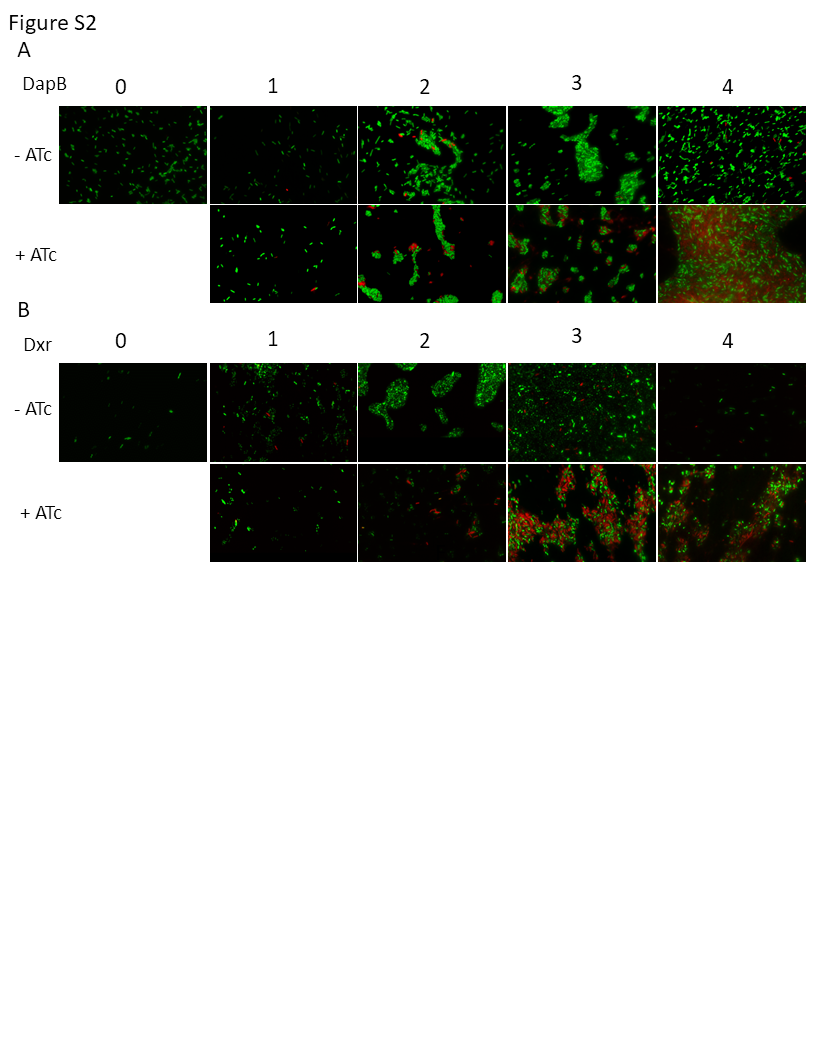

Supplement: FIG S2 [file mbio.03756-21-sf002.tif]
