# Supplementary material for: Degradation of the Escherichia coli Essential Proteins DapB and Dxr Results in Oxidative Stress, which Contributes to Lethality through Incomplete Base Excision Repair
Source: mBio. 2022 Feb 8;13(1):e03756-21. doi: 10.1128/mbio.03756-21 (PMC8822343; doi:10.1128/mbio.03756-21)
Supplement: TABLE S2 [file mbio.03756-21-st002.docx]

| **Strains** | **Genotype** | **Reference** |
| --- | --- | --- |
| MG1655 | F- lambda- *ilvG*- *rfb*-50 *rph*-1 | Lab stock |
| EPD005 | DapB-pdt kan | ([1](#_ENREF_1)) |
| EPD034 | Dxr-pdt kan | ([1](#_ENREF_1)) |
| EPD134 | GryA-pdt kan | ([1](#_ENREF_1)) |
| EPD137 | FolC-pdt kan | ([1](#_ENREF_1)) |
| EPD215 | SecY-pdt kan | ([1](#_ENREF_1)) |
| EPD290 | LexA-pdt kan | ([1](#_ENREF_1)) |
| CG301 | DapB-pdt frt | This study |
| CG302 | Dxr-pdt frt | This study |
| CG302 | CG301 ΔmutM ΔmutY | This study |
| CG303 | CG301 pCA24N | This study |
| CG304 | CG301 pMutT | This study |
| CG305 | CG301 ΔrecA | This study |
| CG306 | CG301 ΔrcsA | This study |
| CG307 | CG301 ΔrcsF | This study |
| CG308 | CG302 ΔmutM ΔmutY | This study |
| CG309 | CG302 pCA24N | This study |
| CG310 | CG302 pMutT | This study |
| CG311 | CG302 ΔrecA | This study |
| **Plasmids** |  |  |
| pCA24N | empty vector used in ASKA collection | ([2](#_ENREF_2)) |
| pMutT | ASKA collection MutT | ([2](#_ENREF_2)) |

1. Cameron DE & Collins JJ (2014) Tunable protein degradation in bacteria. *Nature biotechnology* 32(12):1276-1281.

2. Kitagawa M*, et al.* (2005) Complete set of ORF clones of Escherichia coli ASKA library (a complete set of E. coli K-12 ORF archive): unique resources for biological research. *DNA research : an international journal for rapid publication of reports on genes and genomes* 12(5):291-299.
