# Supplementary material for: Degradation of the Escherichia coli Essential Proteins DapB and Dxr Results in Oxidative Stress, which Contributes to Lethality through Incomplete Base Excision Repair
Source: mBio. 2022 Feb 8;13(1):e03756-21. doi: 10.1128/mbio.03756-21 (PMC8822343; doi:10.1128/mbio.03756-21)
Supplement: TABLE S3 [file mbio.03756-21-st003.docx]

| rrsAF | ACCTTACCTACTCTTGACATCCA |
| --- | --- |
| rrsAR | CCCAACATTTCACAACACGAG |
| rpoAF | TAGAACAGCGTACCGACCTG |
| rpoAR | CGAAAGCTTCCAGTTGTTCA |
| cysGF | TTGTCGGCGGTGGTGATGTC |
| cysGR | ATGCGGTGAACTGTGGAATAAACG |
| ldnTF | CTGTTTAGCGAAGAGGAGATGC |
| ldnTR | ACAAACGGCGGCGATAGC |
| mutMF | ACCATGTGGATTTGGTGATG |
| mutMR | ACATTATGCCCTTCCAGCTC |
| mutTF | TGCGGTAGGTATTATTCGCA |
| mutTR | TCCAGTTTATTCGCCATGTG |
| mutYF | CGCTTTCTCTGGGTAAGCAC |
| mutYR | TACAAATCATCGCACCCAAA |
| soxSF | TACTTGCAACGAATGTTCCG |
| soxRF | ACATAACCCAGGTCCATTGC |
| recAF | GACTATCCGGTATTACCCGGC |
| recAR | GGGCCGCAGATGCGA |
| rcsAF | GTATGTTGCGAATGTGGATGG |
| rcsaAR | GATGAAACGGTCTTGGCTTTG |
| gadEF | GAACAACGATTCGGACAAG |
| gadER | CTCTCCTTTAGTAATCACCGG |
| cadCF | GGTTGTGGCAATTATCATTGC |
| cadCR | GCCGCAACATATTATACCAACAG |
| adiYF | GAGAAATTACTATAAAAATTTGTACTATTAG |
| adiYR | CCTGCGAAGGCGAGCTTC |
| speFF | CGGCACGATCGATTTCTCATTC |
| speFR | GGAGCCCATCATGTTGACCATC |
| hdeAF | TGCGGTTTTAGATGTTCAGGG |
| hdeAR | TTCTTAATTTTGTCCCATTCGCC |
| narHF | GTTATCTTGGCGTGCTGTTG |
| narHR | ATGACTTTCGGATCGTTCGG |
| napAF | TCCTGTTTATTCACCCGCTG |
| napAR | AATCGAGATCACTTCGCCAC |
| dmsAF | ACCGCCTGAAATATCCGATG |
| dmsAR | ACTCGTTGCCGTACTCTTTG |
| nirBF | CGTACACCTCTCGTCTTACTTC |
| nirBR | CCTGACGGTTGATGGTGATAG |
| entBF | GCAAACAGCACAATATCCCG |
| entBR | CCCACATATCATTCAACAGCG |
| feo1F | TTCTCTTGGCATGTTACCTGG |
| feo1R | TTGCGTAATACCAGGCTCAC |
| fhuAF | TGCTCAATCTGTACAATCCGG |
| fhuAR | TGACCAGCACTTTATCCCAC |
